# Supplementary figures and images for: Mutation resource of Samba Mahsuri revealed the presence of high extent of variations among key traits for rice improvement
Source: PLoS One. 2021 Oct 20;16(10):e0258816. doi: 10.1371/journal.pone.0258816 (PMC8528289; doi:10.1371/journal.pone.0258816)

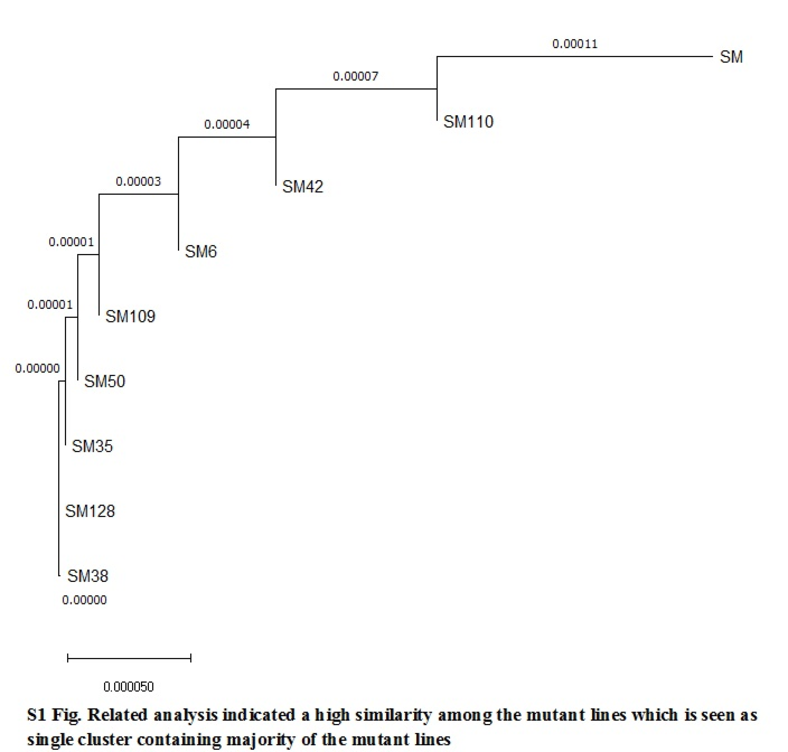

Supplement: S1 Fig — (TIF) [file pone.0258816.s014.tif]
