# Supplementary material for: Mutation resource of Samba Mahsuri revealed the presence of high extent of variations among key traits for rice improvement
Source: PLoS One. 2021 Oct 20;16(10):e0258816. doi: 10.1371/journal.pone.0258816 (PMC8528289; doi:10.1371/journal.pone.0258816)
Supplement: Attachment — Submitted filename: PONE-D-21-18858.pdf [file pone.0258816.s016.pdf]

PLOS ONE

Mutation resource of Samba Mahsuri revealed the presence of high extent of variations among key traits for rice improvement

--Manuscript Draft--

**Manuscript Number:**	PONE-D-21-18858
**Article Type:**	Research Article
**Full Title:**	Mutation resource of Samba Mahsuri revealed the presence of high extent of variations among key traits for rice improvement
**Short Title:**	EMS induced mutants of Samba Mahsuri
**Corresponding Author:**	Maganti Sheshu Madhav, Ph.D.<br>ICAR - IIRR: Indian Institute of Rice Research<br>Hyderabad, INDIA
**Keywords:**	Rice, Samba Mahsuri, Mutagenesis, Mutational breeding and EMS
**Abstract:**	To create novel variants for morphological, physiological, and biotic stress tolerance traits, induced mutations were created using Ethyl Methane Sulphonate (EMS) in the background of Samba Mahsuri (BPT 5204), a popular and mega rice variety of India. A population derived from 10, 500 M 1 plants and their descendants were phenotyped for a wide range of traits leading to the identification of 124 mutants having variations in key agro-morphological traits, and 106 mutants exhibiting variation for physiological traits. Higher yield is the ultimate goal of crop improvement and we identified 574 mutants having higher yield compared to wild type by having better yield attributing traits. Further, a total of 50 mutants showed better panicle exsertion phenotypes as compared to Samba Mahsuri leading to enhancement of yield. Upon rigorous screening for three major biotic stresses, 8 mutants showed enhanced tolerance for yellow stem borer (YSB), and 13 different mutants each showed enhanced tolerance for sheath blight (ShB) and bacterial leaf blight (BLB), respectively. In addition, screening at multiple locations that have diverse field isolates identified 3, 3, and 5 lines for tolerance to ShB, YSB and BLB, respectively. On the whole, 1231 desired mutant lines identified at M 2 were forwarded to an advanced generation (M 5). PCR based allele mining indicated that the BLB tolerant mutants have a different allele than the reported alleles for well-known genes affecting bacterial blight resistance. Whole genome re-sequencing revealed substantial variation in comparison to Samba Mahsuri. The lines showing enhanced tolerance to important biotic stresses (YSB, ShB and BLB) as well as several economically important traits are unique genetic resources which can be utilized for the identification of novel genes/alleles for different traits. The lines which have better agronomic features can be used as pre-breeding lines. The entire mutant population is maintained as a national resource for genetic improvement of the rice crop.
**Order of Authors:**	Gopi Potupureddi
	Vishalakshi Balija
	Suneel Ballichatla
	Gokulan CG
	Komal Awalellu
	Swathi Lekkala
	Karteek Jallipalli
	Gayathri MG
	Ershad Mohammad
	Milton M
	Srikanth Arutla
	Rajender Burka

Powered by Editorial Manager® and ProduXion Manager® from Aries Systems Corporation

	Laha Gouri Shankar
	Padmakumari Ayyangari Phani
	SubbaRao Lella Venkata
	Sundaram Raman Meenakshi
	Viraktamath BC
	Ravindra Babu Vemuri
	Kranthi Brahma
	Raju Madnala
	Hitendra Kumar Patel
	Ramesh Venkata Sonti
	Maganti Sheshu Madhav, Ph.D.
**Additional Information:**	
**Question**	**Response**
**Financial Disclosure**<br><br>Enter a financial disclosure statement that describes the sources of funding for the work included in this submission. Review the submission guidelines for detailed requirements. View published research articles from *PLOS ONE* for specific examples.<br><br>This statement is required for submission and **will appear in the published article** if the submission is accepted. Please make sure it is accurate.<br><br>**Unfunded studies**<br>Enter: *The author(s) received no specific funding for this work.*<br><br>**Funded studies**<br>Enter a statement with the following details:<br>• Initials of the authors who received each award<br>• Grant numbers awarded to each author<br>• The full name of each funder<br>• URL of each funder website<br>• Did the sponsors or funders play any role in the study design, data collection and analysis, decision to publish, or preparation of the manuscript?<br>• **NO** - Include this sentence at the end of your statement: *The funders had no role in study design, data collection and analysis, decision to publish, or preparation of the manuscript.*<br>• **YES** - Specify the role(s) played.	Council of Scientific and Industrial Research (CSIR), New Delhi, India and ICAR -IIRR

* typeset	
**Competing Interests**<br><br>Use the instructions below to enter a competing interest statement for this submission. On behalf of all authors, disclose any competing interests that could be perceived to bias this work—acknowledging all financial support and any other relevant financial or non-financial competing interests.<br><br>This statement is required for submission and **will appear in the published article** if the submission is accepted. Please make sure it is accurate and that any funding sources listed in your Funding Information later in the submission form are also declared in your Financial Disclosure statement.<br><br>View published research articles from *PLOS ONE* for specific examples.<br><br>**NO authors have competing interests**<br><br>Enter: *The authors have declared that no competing interests exist.*<br><br>**Authors with competing interests**<br><br>Enter competing interest details beginning with this statement:<br><br>*I have read the journal's policy and the authors of this manuscript have the following competing interests: [insert competing interests here]*<br><br>* typeset	NO authors have competing interests
**Ethics Statement**<br><br>Enter an ethics statement for this submission. This statement is required if the study involved:	N/A

- Human participants
- Human specimens or tissue
- Vertebrate animals or cephalopods
- Vertebrate embryos or tissues
- Field research

Write "N/A" if the submission does not require an ethics statement.

General guidance is provided below. Consult the submission guidelines for detailed instructions. **Make sure that all information entered here is included in the Methods section of the manuscript.**

Powered by Editorial Manager® and ProduXion Manager® from Aries Systems Corporation

**Format for specific study types**<br><br>**Human Subject Research (involving human participants and/or tissue)**<br>• Give the name of the institutional review board or ethics committee that approved the study<br>• Include the approval number and/or a statement indicating approval of this research<br>• Indicate the form of consent obtained (written/oral) or the reason that consent was not obtained (e.g. the data were analyzed anonymously)<br><br>**Animal Research (involving vertebrate animals, embryos or tissues)**<br>• Provide the name of the Institutional Animal Care and Use Committee (IACUC) or other relevant ethics board that reviewed the study protocol, and indicate whether they approved this research or granted a formal waiver of ethical approval<br>• Include an approval number if one was obtained<br>• If the study involved *non-human primates*, add *additional details* about animal welfare and steps taken to ameliorate suffering<br>• If anesthesia, euthanasia, or any kind of animal sacrifice is part of the study, include briefly which substances and/or methods were applied<br><br>**Field Research**<br><br>Include the following details if this study involves the collection of plant, animal, or other materials from a natural setting:<br>• Field permit number<br>• Name of the institution or relevant body that granted permission	
**Data Availability**<br><br>Authors are required to make all data underlying the findings described fully available, without restriction, and from the time of publication. PLOS allows rare exceptions to address legal and ethical concerns. See the PLOS Data Policy and FAQ for detailed information.	Yes - all data are fully available without restriction

A Data Availability Statement describing where the data can be found is required at submission. Your answers to this question constitute the Data Availability Statement and **will be published in the article**, if accepted.<br><br>**Important:** Stating 'data available on request from the author' is not sufficient. If your data are only available upon request, select 'No' for the first question and explain your exceptional situation in the text box.<br><br>Do the authors confirm that all data underlying the findings described in their manuscript are fully available without restriction?	
**Describe where the data may be found in full sentences. If you are copying our sample text, replace any instances of XXX with the appropriate details.**<br><br>• If the data are **held or will be held in a public repository**, include URLs, accession numbers or DOIs. If this information will only be available after acceptance, indicate this by ticking the box below. For example: *All XXX files are available from the XXX database (accession number(s) XXX, XXX.).*<br>• If the data are all contained **within the manuscript and/or Supporting Information files**, enter the following: *All relevant data are within the manuscript and its Supporting Information files.*<br>• If neither of these applies but you are able to provide **details of access elsewhere**, with or without limitations, please do so. For example:<br><br>*Data cannot be shared publicly because of [XXX]. Data are available from the XXX Institutional Data Access / Ethics Committee (contact via XXX) for researchers who meet the criteria for access to confidential data.*<br><br>*The data underlying the results presented in the study are available from (include the name of the third party*	All relevant data are within the manuscript and its Supporting Information files.

and contact information or URL).<br>• This text is appropriate if the data are owned by a third party and authors do not have permission to share the data.<br><br>* typeset	
Additional data availability information:	

Manuscript
Click here to access/download;Manuscript;MS_Plosone_mutants.doc

Mutation resource of Samba Mahsuri revealed the presence of high extent of variations among key traits for rice improvement

Short title: EMS induced mutants of Samba Mahsuri

Gopi Potupureddi[1#], Vishalakshi Balija[1#], Suneel Ballichatla[1#], Gokulan CG[#3], Komal Awalellu[3], Swathi Lekkala[1], Karteek Jallipalli[1], Gayathri MG[1], Ershad Mohammad[1], Milton M[1], Srikanth Arutla[1], Rajender Burka[1], Laha Gouri Shankar[2], Padmakumari Ayyangari Phani[2], SubbaRao Lella Venkata[1], Sundaram Raman Meenakshi[1], Viraktamath BC[1], Ravindra Babu Vemuri[1], Brahma Kranthi[3], Raju Madnala[3], Hitendra Kumar Patel[3], Ramesh Venkata Sonti[3*] and Sheshu Madhav Maganti[1*]

1: Crop Improvement, ICAR- Indian Institute of Rice Research, Hyderabad, India

2: Crop Protection, ICAR- Indian Institute of Rice Research, Hyderabad, India

3: CSIR-Centre for Cellular and Molecular Biology, Hyderabad, India

# Authors contributed equally

*** Corresponding Authors:**

1. Maganti Sheshu Madhav,

Principal Scientist, Department of Biotechnology,

ICAR-Indian Institute of Rice Research,

Rajendranagar, Hyderabad-500030, India.

Email: sheshu24@gmail.com

2. Ramesh Venkata Sonti

J.C. Bose Fellow

CSIR-Centre for Cellular and Molecular Biology,

Hyderabad-500007, India, Email: sonti@ccmb.res.in,

Keywords: Rice, Samba Mahsuri, Mutagenesis, Mutational breeding and EMS

Abstract: To create novel variants for morphological, physiological, and biotic stress tolerance traits, induced mutations were created using Ethyl Methane Sulphonate (EMS) in the background of Samba Mahsuri (BPT 5204), a popular and mega rice variety of India. A population derived from 10, 500 M_1 plants and their descendants were phenotyped for a wide range of traits leading to the identification of 124 mutants having variations in key agro-morphological traits, and 106 mutants exhibiting variation for physiological traits. Higher yield is the ultimate goal of crop improvement and we identified 574 mutants having higher yield compared to wild type by having better yield attributing traits. Further, a total of 50 mutants showed better panicle exsertion phenotypes as compared to Samba Mahsuri leading to enhancement of yield. Upon rigorous screening for three major biotic stresses, 8 mutants showed enhanced tolerance for yellow stem borer (YSB), and 13 different mutants each showed enhanced tolerance for sheath blight (ShB) and bacterial leaf blight (BLB), respectively. In addition, screening at multiple locations that have diverse field isolates identified 3, 3, and 5 lines for tolerance to ShB, YSB and BLB, respectively. On the whole, 1231 desired mutant lines identified at M_2 were forwarded to an advanced generation (M_5). PCR based allele mining indicated that the BLB tolerant mutants have a different allele than the reported alleles for well-known genes affecting bacterial blight resistance. Whole genome re-sequencing revealed substantial variation in comparison to Samba Mahsuri. The lines showing enhanced tolerance to important biotic stresses (YSB, ShB and BLB) as well as several economically important traits are unique genetic resources which can be utilized for the identification of novel genes/alleles for different traits. The lines which have better agronomic features can be used as pre-breeding lines. The entire mutant population is maintained as a national resource for genetic improvement of the rice crop.

Introduction:

Rice is the staple food for almost 56% of the world's population and it is the source of 20% of the world's dietary energy supply. Global rice consumption is projected to increase from 500 million to around 750 million tons by 2050 (Pathak et al. 2018). Hence, rice production has to be enhanced (50% projected growth) to match the increased requirements of the future. Development of genetic stocks suitable for various agro-climatic zones is being done based on selection among the improved cultivars or through bringing improvements of mega varieties by modifying specific traits. This is often leading to a narrow genetic base which results in low variability and consequently limits the possibilities for recombination and genetic segregation, leading to reduced genetic gains (Fu, 2015). Therefore, in spite of the development and release of numerous rice varieties and hybrids, the genetic gain has been relatively low (Singh et al. 2016). Hence, there is a need to enhance available variation amongst the rice germplasm. Induced mutations are one of the best available options to create variation among the well adapted mega varieties. Mutations induced artificially using physical agents like fast neutron (Ruengphayak et al. 2015), γ-ray (Lie-bao et al. 2005; Morita et al. 2009), ion beam (Lee et al. 2015) and chemical mutagens such as ethyl methane sulfonate (EMS) (Lee et al. 2003, Till et al. 2007, Mohapatra et al. 2014), methyl nitrosourea (MNU) (Suzuki et al. 2008), sodium azide (SA) (Awan et al. 1980 and Tai et al. 2016) have been employed in rice to create mutations. Chemical or physical mutagens have higher mutation efficiencies as compared to insertions created by T-DNA (Ram et al. 2019), transposable elements (Greco et al. 2001, Xuan et al. 2016) or RNAi (Wang et al. 2013), TALEN-based gene editing (Li et al. 2012), and CRISPR/Cas9 genome editing (Xie et al. 2015).

Among the mutagens, EMS is being used frequently owing to its production of high frequency (2 to10 mutations/ Mb) of single nucleotide changes (point mutations) by alkylation of specific nucleotides (Till et al. 2007; Viana et al. 2019) and a relatively small population, ca. 10,000 plants is sufficient to saturate the genome with mutations. According to FAO/IAEA-MVD reports (2020) (www.mvd.iaea.org), a total of 828 mutant varieties were developed in rice among them 60 were derived through chemical mutagenesis. Till date using chemical mutagens, 6 mutant resources in diverse *Japonica* cultivars (Nipponbare, Tainung 67, Kitaano, TC65, Yukihikari, Kinmaze, and BRS Querencia), three mutant resources in *indica* cultivars (IR64, Kasalath, and SSBM) and one *aus* variety (Nagina22) were developed (Li et al. 2017). The International Rice Functional Genomics Consortium announced the public availability of more than 200,000 rice mutant lines, which represent mutations in about half of the known functional genes mapped for rice to date (Krishnan et al. 2009). In India, one EMS mutant resource was developed in the genetic background of Nagina 22 (N22) which is a drought and heat tolerant *aus* rice variety (Mohapatra et al. 2014. Using this mutational resource, Shoba et al. (2017) identified a herbicide tolerant mutant (HTM-N22.)

Samba Mahsuri (BPT-5204) is a mega rice variety of India, released in the year 1984 and is now also grown in other Asian countries. It is a long duration (145-150 days) fine-grain type variety, yielding an average of 5-6 t ha $^{-1}$ with excellent cooking quality and has wide adaptability. However, this variety is susceptible to biotic stresses like sheath blight, blast, bacterial leaf blight (BLB) and insect pests such as yellow stem borer (YSB), brown plant hopper (BPH) and gall midge. In spite of these constraints, it is favored by consumers and farmers in India, because of its excellent cooking and eating quality characteristics. Enhancing genetic variability in Samba Mahsuri would be useful to tackle some of the existing constraints of this

variety. Furthermore, the excellent combining ability of this variety (Sundaram 2018) will help in transferring such traits into other varietal backgrounds. Because of the above features, Samba Mahsuri is an ideal genotype to develop a comprehensive mutant population.

Currently, mutagenesis coupled with molecular mapping and development of molecular markers has become an important tool with excellent potential for crop improvement (Kharkwal and Shu, 2009). Markers identified through next-generation sequencing technologies (NGS) are an efficient and cost-effective approach to characterize the variants in mutant collections (McCallum et al. 2000). Mutant resources are being used not only for creating variation but also for novel gene identification and deciphering gene function (Xu et al. 2017). To generate variability in the background of Samba Mahsuri, the current research focused on developing mutants using EMS and characterized them for various morphological, physiological, yield traits and biotic stress tolerance so as to use these mutants as donors in rice improvement programs.

Materials and Methods

Mutagenesis of rice seeds

The rice variety, Samba Mahsuri (Nucleus seed obtained from Bapatla Rice Research station, Andhra Pradesh, India) was used for mutagen treatment. Based on kill curve analysis the seeds were treated in two different concentrations of Ethyl methanesulphonate (EMS) *i.e.*, 1.2% (1-6819) and 0.8 % (6820-10500) by M/s Bench Biotech Company, Gujarat, India. The duration of treatment was about 12 hrs and the treated seeds were washed thoroughly under running tap water for 6 h to leach out the residual chemical.

Development of M_1 and M_2 generations

A total of 10,500 M_1 plants were raised from mutagenized (M_0) seeds in the fields of M/s Bench Biotech. These M_1 plants were protected from out crossing through bagging and harvested individually to obtain the M_2 seeds from main panicles. The collected M_2 seeds were distributed among the participating centers CSIR-Centre for Cellular and Molecular Biology (CSIR-CCMB) and ICAR-Indian Institute of Rice Research (ICAR-IIRR) for further studies. The M_2 seeds were raised at ICAR-IIRR and International Crop Research Institute for Semi-Arid Tropics (ICRISAT), Patancheru, Hyderabad during kharif (June-July to October-November) 2013.

Phenotypic characterization of the Samba Mahsuri mutants

A large subset of 10,500 M_2 lines were subjected to various morphological (Plant height, Pink apicules, Albino, Xantha and Coloration), agronomical (panicle types, complete panicle emergence, early flowering, grain types and high yield with high grain number), physiological (strong culm, different types of flag leaf, sterile plant, stay green and shattering) and biotic stresses (sheath blight, bacterial leaf blight and yellow stem borer) studies.

Plant height was measured from the base of the plant to the tip of the tallest panicle. High yielding mutants were phenotyped based on the yield parameters like number of tillers, number of panicles, panicle length (cm), grain number per panicle and yield per plant (g) at maturity (Singh et al. 2018). The phenotypic acceptability of mutants was also measured; the mutants having optimum number of tillers at 4-5 leaf stage were selected. The number of days from seeding to grain ripening (95% of grains on panicle are mature) was recorded and maturity duration of the mutants was observed. The lines that matured earlier than the wild type (Samba Mahsuri) were selected as early maturing mutants. Mutants having length of > 2cm of uppermost internode were selected as EUI (Elongated Upper Internode) mutants (Xu et al. 2004). The mutants which showed complete emergence of panicle not having any choking in the flag leaf

but not showing the elongated upper internode were considered as complete panicle emergence (CPE) mutants. The grain lengths were measured by using a digital Vernier caliper (M/s Mitutoyo, USA) and classified based on length and breadth of grain (Ramkumar et al. 2010). For the identification of strong culm mutants, the diameter and physical strength of the culm were taken into account. Culm diameter was measured using a digital sliding Vernier caliper in the field from the plants at a height of 30cm above ground level (Yadav et al. 2017), while the physical strength of the culms was measured with a 'prostrate tester' (DIK-7401 Daiki Rika Kogyo co Ltd, Tokyo, Japan) where the plant was pushed at an angle of 45° to calculate the pushing resistance (Hai et al. 2005).

In each generation, the mutants with the trait of interest were identified and advanced to the next generations in ear to row method. The whole mutant population was advanced up to M_4 generation considering the diversity. Later on, from M_5 generation, the advancement of mutants was done based on yield and uniformity. The data for the above-mentioned traits were recorded at three different crop growth stages *i.e.*, seedling, vegetative, and reproductive stages following DUS guidelines (Mohapatra et al. 2014).

Screening of mutant lines for biotic stresses

Screening for Yellow Stem Borer (YSB) resistance/susceptibility was done in a phased manner at both vegetative and reproductive phases by augmenting the natural pest infestation through artificial releases (Devasena et al. 2018). The susceptibility and resistant level of the mutant lines was determined based on the Standard Evaluation Scale (SES) scale, IRRI, 2014. The varieties, Pusa Basmati-1, and Samba Mahsuri were used as susceptible checks, while W1263 was the tolerant check for YSB screening. The selected lines were further evaluated in

multi location stem borer screening trial under AICRIP (All India Coordination Rice Improvement Programme)- Entomology programme for two seasons (2016 and 2017).

Screening for sheath blight tolerance/susceptibility of the mutant population was done at two locations *viz.*, ICAR-IIRR and ICRISAT farms using a highly virulent isolate of rice sheath blight pathogen *R. solani* (WGL-12 isolate) as well as hotspot locations of India using virulent isolate of that location. Artificial inoculation of the isolate was done in colonized typha stem bits and culture prepared according to the procedure given by Bhaktavatsalam et al. (1978). Varieties TN1 and Samba Mahsuri (Wild type) were used as susceptible checks, while 'Tetep' was used as a tolerant check for sheath blight screening. Observations were recorded after 20 days of inoculation and scored as per IRRI-SES scale (Standard Evaluation System) (IRRI, 1996). Further, the selected lines which showed tolerance to sheath blight under field and laboratory conditions were screened with 10 diverse *R. solani* isolates (*viz.,* TN14-1, RNR 13-F, TTB-1, WGL-12-1, Gosaba-1, Kaual, PNT, Jamalpur- Bangar, and Imphal-1) collected from various hotspot regions of India. The three best mutant lines were again screened at four hotspot locations (Kaul, Pantnagar, Chinsura and MCP) in India for further confirmation of sheath blight tolerance.

Similarly, the mutagenized population was evaluated for bacterial leaf blight (BLB) under artificial conditions following the clip-inoculation method (Kauffman et al, 1973). The lesion lengths were recorded after 15 days of infection and scored according to SES scale, IRRI (2014) (Yugander et al. 2019). For BB screening, the varieties TN1 and Samba Mahsuri (wild type parent) were used as susceptible checks, Improved Samba Mahsuri (a derivative of Samba Mahsuri having *Xa21, xa13* and *xa5* genes; Sundaram et al, 2008) was used as the resistant check. The identified resistant mutants were further screened with multiple *Xoo* pathotypes.

These pathotypes included LUD-05-1 (IXo-090; compatible for *xa5*, *xa13* and *Xa21*), ADT (IXo 015; compatible for *xa5* and incompatible for *xa13* and *Xa21*), TNK-12-3 (IXo-281; compatible for *xa5*, *xa13* and incompatible for *Xa21*), CHN/J (IXo-027; compatible for *Xa21*, *xa5* and incompatible for *xa13)* and DRR-1 (IXo-020) pathotype (compatible for all the three genes *xa5*, *xa13* and *Xa21).*

Assessment of BLB tolerant mutants for allelic status of known resistance genes, *Xa21*, *xa13* and *xa5*

The bacterial leaf blight (BLB) tolerant lines were screened for allelic variations among the reported genes for BLB resistance (namely *Xa21*, *xa13* and *xa5*) through PCR based screening technique. The DNA extraction was carried out by modified CTAB method (Saghai-Maroof et al. 1984). The primer details of BLB resistance genes (*Xa21, xa13* and *xa5*) are provided as Table S1. The PCR reaction volume and profile was followed using the protocol of Hajira et al., 2016.

Whole genome re-sequencing of mutants

The genomic DNA was isolated from three genetic stocks (which include the nucleus seed of Samba Mahsuri which was used for mutagenesis along with two other stocks of this variety) as well as eight mutants with different traits using the CTAB method as mentioned above. The isolated genomic DNA was checked for quality on an agarose gel and quantity using Qubit 4 fluorometer (Thermo Fisher Scientific, USA). Library preparation and sequencing was outsourced to Nucleome Informatics Private Limited, Hyderabad, India. In short, sequencing libraries were prepared using Truseq Nano DNA HT Sample preparation Kit (Illumina, USA) and qualified libraries were sequenced on Illumina HiSeq2500 platform to obtain 2x150 bp reads at approximately 40X coverage.

The obtained high-quality reads from the three genetic stocks of Samba Mahsuri were used for constructing the Samba Mahsuri reference genome using the consensus calling approach described previously (Abe et al. 2012). Briefly, the sequenced reads were aligned using *bwa mem* (v0.7.17; http://bio-bwa.sourceforge.net/bwa.shtml) to the *indica* reference genome, R498 (Du et al. 2017). Using *Samtools* (v0.1.20; http://www.htslib.org/doc/samtools.html) the bam files were sorted, and deduplicated. The final bam files were used for variant calling with reference to R498 genome using *freebayes* (v1.0.0; Garrison et al. 2012). The variants were filtered using *VCF tools* (v0.1.15; Danecek et al. 2011) and bash scripts to retain only homozygous SNPs supported by a minimum of 10 reads and with minimum quality score of 30. All the SM sequences were processed in the same way and finally all the SNPs were merged to obtain a single VCF file. Using *BCF tools* (v1.7; http://samtools.github.io/bcftools/bcftools.html) *consensus*, the SNPs were replaced in the R498 reference genome to obtain an SM reference genome.

The Samba Mahsuri reference genome was used for aligning the mutant sequence reads and variant calling using the same pipeline as discussed above. After obtaining the filtered SNPs, only the GC to AT type transitions were retained for further analyses. Custom scripts were used for identifying the chromosome-wise distribution of the SNPs. Dissimilarity index was calculated by dividing the total number of GC to AT type homozygous SNPs by the total number of bases in the reference genome (i.e., 390,983,850). *Tassel 5* was used for obtaining the relatedness dendrogram (Bradbury et al. 2007). SnpEff (v4.3t; Cingolani et al. 2012) was used for annotating the SNPs.

Results

A total of 10,500 mutants (M_1) were generated upon treatment of Samba Mahsuri with EMS mutagen in two different concentrations. Phenotypic observations at M_2 generation for various morphological, physiological, and yield parameters identified 1231 putative mutants (~ 12% of population) showing promising variation for the traits of interest (Table 1; S2 to S7 Table). These mutants were categorized for different traits, among those, the lines showing variation in the yield contributing traits occupied the major proportion (43%), followed by physiological and panicle emergence mutants (15% each) and the panicle type and morphological trait mutants (Fig 1). The selected mutants were subsequently advanced to M_3 and M_4 generations wherein 418 and 276 mutants showed stable inheritance of variations, respectively (S2 to S7 Table). Considering the traits of interest, 180 mutants were selected and advanced to M_5 generation for further studies (S2 to S7 Table).

Upon screening of mutagenic population in M_2 generation for three biotic stresses (YSB, ShB, and BLB), 1453 promising mutant lines were identified, of which ShB mutants occupied the major proportion (55%). These mutants were advanced to the subsequent generations and a total of 34 stable mutants were recovered in M_5 generation for the three biotic stresses (Table S8 to S10).

Variations in the morphological traits

Around (0.79%, 83 mutants) of the total population showed variation in various morphological traits (viz., tall, dwarf, grassy bonsai and pink apiculus) (Fig. 2A & 2B). Among these, mutants having tallness (130-155cm) were the highest proportion (51%, 43 mutants) of the total morphological traits, followed by dwarf mutants (43.3%; 36 mutants) with the height being less than 100cm. We also identified one extreme dwarf mutant (bonsai type having a shoot

length of 35cm). This mutant had less number of internodes (n=3) and very narrow internodal length with grassy appearance. The details of the traits and the number of mutants observed in each generation are given in Table 1 and Table S2.

Variations in the physiological traits

The M_2 mutants were characterized for various physiological traits *viz.*, flag leaf variation, leaf variations, albino, xantha, coloration, strong culm, stay green, early maturity, shattering and sterility. Of the total population, 1.8% mutants (191) showed variations in the physiological traits. Among these, strong culm that imparts lodging resistance is an important trait and we identified 26 mutants (0.24%) with culm strength ranging between 26-32 N/m^2, while the wild type showed culm strength of 25N/m^2 (Fig 2C). Early maturing (108-132 days) mutants were observed at a frequency of 0.69% (73 mutants) (Fig 2D). Stay-green in the post-anthesis period is found to be an efficient drought tolerance trait and we obtained 4 stay green mutants (0.03%) in the M_2 generation. We also obtained a good frequency (0.26%) of sterile plants; however, these could not be maintained further due to non-uniform segregation (Table S3).

Variations in panicle types

Panicle type variants (sparse, long, and dense) were observed at a proportion of 0.8% (85) of the total M_2 population. Among these, long panicle (30-32cms) type mutants were the highest with 0.47% (50 mutants) (Fig 2E). The dense panicle mutants recorded 300-350 seeds per plant, while the wildtype had only 180-200 seeds (Fig 2F; Table S4).

Variations in panicle emergence

Complete panicle emergence (CPE) and Elongated Upper Internode (EUI) are two major traits that were studied under the category of panicle emergence. In this category, we identified 182 mutants (1.72% of the population) of which CPE mutants were more in number (1.47% of

the population) and the EUI mutants were less frequent (0.25% of the population). The CPE mutants exhibited 2.0 to 2.5cm of panicle exsertion and thus no panicle choking (Fig 2G) whereas 10-20% panicle chocking was observed in wild type. The EUI mutants exhibited a length of 7-26cm for the upper most internode (Fig 2H) (Table S5).

Variations in yield and yield attributing traits

The yield attributing traits included more number of tillers, number of productive tillers and better grain filling, grain number and phenotypic acceptability (PA). In the M_2 generation, the mutants in yield traits were the most frequent; *i.e.* a total of 532 mutants representing 5.06% of the total mutant population (Fig. 2I). Among these traits, number of productive tillers and better grain filling occupied the major proportion with 4.06% (484 mutants), while more number of tillers (15-26 tillers) occupied the least with a proportion of 0.12%. Overall phenotypic acceptability of the mutant lines was also recorded and we obtained a frequency of 0.19% (20 mutants) in the M_2 generation (Table S6).

Variations in the grain types:

The wild type has medium slender grain type, however upon treatment with EMS we observed a wide range of grain types (Table 1). The grain type mutants constituted 1.5% (158) of the total M_2 population, of which medium bold grain without awns were the highest frequency (0.36%), followed by long slender grains without awns (0.34%). Interestingly, we also observed seven (0.06%) sickle shaped seed mutants. The types of variants obtained are indicated in Fig. 2J. All these mutants were forwarded to M_5 generation, however in subsequent generations we focused on medium slender and long slender grain types, as these are the commercially preferred grain types (Table S7).

Biotic stress variation among the mutants

The mutant population that had been generated was large and screening such a large population at one time for various biotic stresses in the same season was not practical. For ease of operations, 2500 M_2 families each were screened in two seasons for all three biotic stresses. While screening in the second season, the selected tolerant mutants from the first season were carried forward for rescreening in the next generation along with the base mutant population. A high frequency (13.83%) of the total population showed tolerance for at least one of the three biotic stresses at M_2 generation. But after rescreening till M_5 generation, the frequency of tolerant lines reduced from 13.83% to 0.33%. At the M_2 generation, sheath blight tolerant mutants were the most frequent (7.62%) followed by the bacterial leaf blight tolerant lines (4.59%) and the YSB tolerant lines (1.61%) (S8 to S10 Table). The reduction in frequency of tolerant mutants following rescreening indicates that there were a large number of false positives possibly due to lines that escaped infection. The details of the putative tolerant mutants obtained in each generation for various biotic stresses are given in Table 2 and the visual symptoms are given in fig 2K, 2L and 2M.

Characterization of mutants at multi locations for biotic stress

Among 13 sheath blight mutants, three mutant lines (SB-5, SB-6, and SB-8) showed high level of resistance with a mean-scores of 2.84, 3.24 and 3.28 whereas BPT 5204 (wild type), TN-1 and resistant control (Tetep) showed score of 9, 9 and 4.45, respectively (Table S11). Further, these three best mutant lines revealed the similar level of tolerance at four hotspot locations of India. This indicates that these three lines are extremely good sources of tolerance for sheath blight. One of the sheath blight mutant (Sb-5) is registered recently with National bureau of plant genetic resources (NBPGR, New Delhi) as INGR 20080 (available at

http://www.nbpgr.ernet.in:8080/registration/ApplicationList.aspx). A total of 13 lines showed consistent resistance reaction for bacterial blight under field conditions. Among them 5 (BB-42, BB-49, BB-61, BB-132 and BB-133) lines showed resistance level ranging from mean score 1 to 3 to all the tested pathotypes, indicating that the selected mutants were resistant to multiple isolates of the pathogen in India (Table S11) whereas the wildtype (Samba Mahsuri), resistant (Improved Samba Mahsuri) and susceptible (TN-1) checks exhibited mean scores of 7.4, 1.4, and 9 respectively.

The eight mutants identified to be tolerant to YSB were evaluated under AICRIP trials for two seasons. Among them, two mutants i.e., IIRR-Bio-SB-9/SM-92 (20.48 and 20.04g/hill) and IIRR-Bio-SB-5/SM-48 (17.22 and 20.64 g/hill) were most promising exhibiting tolerance to YSB with more grain yield/ hill compared to wildtype (16.92 and 17.15g/hill) and tolerant check, W1263 (16.56 and 19.75g/hill) in two seasons (Table S11).

Mutants with multiple trait variations

Most of the mutants exhibited trait-specific variations in comparison with Samba Mahsuri, but a few mutant lines (n=8) showed variation in multiple traits. For instance, the mutant line TI -24 showed different trait combinations like strong culm, early flowering, medium bold, pink apiculus, complete panicle emergence, broad flag leaf and dense panicle and stay green. Similarly, another mutant TI-21 also exhibited variations in multiple traits *viz.*, strong culm, broad flag leaf and early flowering yet showed medium slender grain like wild type. The details of the mutants which showed multiple trait variations are given in the Table S12.

The alleles contributing to tolerance in BLB tolerant lines are different from the *Xa21, xa13* and *xa5* resistance alleles that have been deployed in resistance breeding programs in India:

In recent years, the BLB resistance genes *Xa21, xa13* and *xa5* have been frequently used in resistance breeding programs in India. Therefore, the allelic status of these five BLB tolerance lines (BB-42, BB-49, BB-61, BB-132 and BB-133), was assessed with gene specific (*Xa21, xa13* and *xa5*) markers. The positive check was Improved Samba Mahsuri (ISM) which has the resistance alleles at all three loci and the susceptible checks were TN1 and Samba Mahsuri (wild type) rice varieties which have the susceptibility alleles at all three loci. The genotyping with gene specific markers indicated that none of the five BLB tolerant lines tested carry the resistance alleles that are present in ISM. Thus, these BLB tolerant lines may have some other genes/alleles that contribute to tolerance.

Validation of dominant/recessive nature of traits:

Selected mutants of strong culm (TI-17) and CPE (CPE-109 and CPE-110) mutants were crossed with wildtype and F_1 progeny were generated. The F_1 progeny from these crosses exhibited a wild type phenotype indicating that the traits are governed by recessive alleles.

Sequencing of mutant lines:

We performed whole genome re-sequencing of the wildtype (three genetic stocks) and eight mutant lines showing different traits to study the extent of mutagenesis. We obtained high quality sequences with an average depth of 41x (Table 3). Greater than 98% of the reads from the mutant lines mapped to the Samba Mahsuri reference genome (Table 3). Analyses of the EMS-induced SNPs revealed that the dissimilarity between Samba Mahsuri and the mutant lines ranges from 0.008% to 0.032% (Table 4). The SNPs/Mb was ranging from 76.3 to 322.3 bases (Table 4). As expected, many more SNPs were present in non-coding regions as compared to the coding regions (Table S13). The high yield mutant TI-128 had the highest number of SNPs whereas the TI-42 line (tolerant to BLB) had the lowest number of SNPs in both coding and non-

coding regions. Annotation of the SNPs showed that 8.86 to 10.01 % of the SNPs were in the coding regions of the mutant lines (S14 Table) and relatedness analysis revealed clustering of the mutant lines (Fig S1). The number of SNPs in the CDs ranged from 2962 (TI-42) to 12268 (TI-128). The number of SNPs in the 3' UTR region were ranging from 1195 (TI-35) to 4855 (TI-128) whereas the number of SNPs in the 5' UTR region ranged from 1147 (TI-42) to 4320 (TI-128). The number of SNPs in the non-coding regions ranged from 24301 (TI-42) to 103796 (TI-128).

Discussion:

Samba Mahsuri (BPT-5204) is a popular rice variety in India because of its excellent cooking and eating qualities but it is highly susceptible to many biotic and abiotic stresses. The everlasting demand for this variety has encouraged various researchers to select this variety for marker assisted selection (MAS) to improve tolerance for some of the stresses, where the donors are available in the gene pool. To enhance the scope of MAS and to develop the donors for traits where the donors are not available, creation of variation through mutagenesis was sought. Among various physical and chemical mutagens, EMS majorly induces point mutations and less chromosomal segment breaks, as well demonstrated in rice (Till et al 2007; Greene et al. 2003). The screening of mutants generated by T-DNA or transposons suffer from restrictions for field evaluation as they are considered as genetically modified (GM) but this restriction does not apply for the EMS induced mutants. Owing to these advantages, in the present study, EMS was opted for induction of mutagenesis. Till date, more than 22 mutant resources were developed in diverse genetic backgrounds of rice using different mutagens across the globe (Sevanthi et al. 2018), but no studies were reported on the development of mutagenic population in the background of Samba Mahsuri.

Identification of suitable mutagenic dose is the key component in developing mutagenic populations since it determines the mutation densities. To generate such population, Samba Mahsuri seed were treated with EMS in two different concentrations (1.2% and 0.8% for 12 hrs) and the 1.2% treatment yielded a greater frequency of mutants with favored agro-morphological traits. Similar mutation induction studies were conducted by Mohapatra et al. (2014) in *aus* cultivar Nagina 22 and confirmed that 1.5% for 12 hrs of EMS treatment generated useful mutants after using a wide range of EMS concentrations (0.2-2%). Wu et al. (2005) established a mutant population in IR64 using five different EMS concentrations (0.4, 0.6, 0.8, 1.0 and 1.6%) for kill curve analysis and observed that the 1.6% concentration exhibited the highest frequency rate of mutations. Talebi et al. (2012) used six different concentrations (0.25%, 0.50%, 0.75%, 1%, 1.25%, 1.5% and 2%) of EMS to treat MR219, a Malaysian *indica* variety and fixed 0.25% and 0.50% for 12 hrs as LD values for generating variation. Several mutagenesis studies on plants have reported that the optimal concentration of the chemical mutagen can vary with the variety used, which might be due to possible influence of the genome on the effects of the mutagen (Rao and Reddi, 1986).

The progeny from a set of 10,500 Samba Mahsuri M_1 mutant lines were characterized for agro-morphological traits and biotic stresses from M_2 to M_5 generations and mutants were identified that have variations in different morphological characters like height (tall, dwarf, bonsai) and pink apiculus. Similar plant height variations were also identified by Mohapatra et al. 2014. Our observations also suggested that some mutants might have mutations with large phenotypic effects which fell well outside the phenotype of the wild type.

The flag leaf and top leaf blades play a major role in photosynthesis and affect the grain yield of rice (Takai et al. 2013). Therefore, we classified mutants having broad and long flag leaf

variations in the category of physiological traits. These mutants might carry mutations among alleles/genes responsible for flag leaf development. Earlier studies demonstrated that a mutation in the Flag Leaf gene (*NAL 1*) affects various yield related traits in rice (Taguchi-Shiobara et al. 2015). Similarly, Sakamoto et al. (2005) explained that the brassino-steroid deficient mutant having erect leaves showed increase in biomass and grain yield in rice. Phenotypes such as albino, xantha and coloration mutants were observed in M_1 generation, but they were not observed in adult plants of M_2 generation indicating that they were lethal.

Improvement of lodging resistance by incorporation of the semi-dwarf trait alone is not sufficient and other traits such as strong culm are needed for reinforcement of the phenotype (Ookawa et al. 2016). According to Ookawa et al. (2010), the diameter and wall thickness will influence the parameter of the physical strength of the culm. A QTL associated with strong culm was earlier identified by Ookawa et al. 2010. Using chromosomal segment substitution lines, they have reported that *Strong Culm 2* (*SCM 2*), a gain-of-function mutant of APO1, was responsible for the strong culm phenotype. The strong culm mutants identified in this work need to be characterized further to identify the genes/alleles that are contributing to this trait. Maturity duration is one of the important growth parameters that determine crop productivity under a given climatic condition. We recovered thirty-six early maturing mutants (maturing between 108-135 days). Mutants that are similar in yield with wild type but having an early maturing nature are very useful for the rice varietal improvement programme. Similarly, Chakrabarti et al. (1995) developed early maturing PNR series mutants utilizing gamma radiations in Basmati 370 PNR-17-3 and Mustikarini et al. (2017), developed a gamma-ray induced red rice (*Celak Madu* accession) mutant which matures earlier than the wild type (*i.e.,* in 115 days) and is drought tolerant.

The stay green or delayed leaf senescence phenotype of the plant is another attractive and desired characteristic of rice. Very few genetic sources are available for this trait and hence the stay green mutants identified in this study may be helpful in improving rice production under stressful environment conditions. In this connection it is pertinent to note that Ramkumar et al. (2019) have identified a stay green mutant (*SGM-3*) of Nagina22 which has better harvest index under drought stress.

Similarly, elongation of the uppermost internode (EUI) is an important component of plant architecture that can be used to overcome the sheathed panicle of the rice male sterile line (Shen et al. 1987) by enhancing pollen dispersal and facilitating hybrid rice seed production (Yang et al. 2002). In our study, the identified EUI mutants were observed to have better upper node elongation than the wild type and their incorporation into the CMS line can help enhance pollen dispersal. Okuno and Kawai (1978) first reported EUI mutants, derived from the Japanese rice cultivar Norin 8 by gamma-ray treatment. Similar EUI studies were conducted by Rutger and Carnahan (1981) and they reported enhanced upper internode elongation during the heading stage. Similarly, incomplete panicle emergence leads to yield reduction (Yuan et al. 1988) which is generally observed in Samba Mahsuri resulting in 10-15% reduction in yield. Hence, the identified mutants that have complete panicle emergence may play an important role in rice breeding programmes to increase yield of Samba Mahsuri as well as other rice varieties whose yield may be affected due to incomplete panicle emergence.

A number of high yielding mutant lines were identified that have more tiller numbers and higher grain numbers. Similarly, Roy et al. (2018) identified eight high yielding aromatic mutants in the background of Tulpanji using gamma irradiation. Soomro et al. (2002) identified two high yielding mutant lines derived from IR6, through gamma rays. Several high yielding

mutants were also popular in many countries such as Vietnam (55 mutant varieties), Bangladesh (44 mutant varieties) and Thailand (two aromatic mutant varieties (RD6, RD15) where they have been cultivated over millions of hectares (Oladosu et al. 2016). In India, the PNR-381 and PNR-102 aromatic rice varieties are early maturing rice mutants that are derived from the Basmati 370 PNR-17-3 cultivar and have become popular in Haryana and Uttar Pradesh States (https://mvd.iaea.org; Chakrabarti, 1995).

In the present study, different grain type mutants were isolated. They could have importance in varietal improvement since the visual characteristics of rice grains is an important attribute that affects consumer's preference. Azad et al. (2012) developed different grain type mutants by irradiating IR8 with gamma rays. Different grain types (short, long, bold) were also reported from the Nagina22 mutant population (Mohapatra et al. 2014). A mutant line derived from Kitake rice variety using fast-neutron (FN) mutagenesis carried a new *grain shape 9–1* (*gs9–1*) allele that also affects grain size (Jiang et al. 2019).

In this study, mutants having variations in multiple traits were observed and such kinds of mutants were also reported earlier in Nagina 22 background (Mohapatra et al. 2014). These mutants may either carry point mutations in multiple genes that independently affect the observed traits or carry a mutation that has a pleiotropic phenotype (Patil, 1966; Till et al. 2007).

In the present study mutation frequency of many mutant types showed a decrease in the number as we progressed from M_2 to M_5. We are unable to explain this apparent reversion phenomenon as the phenotype was quite apparent in the M_2 generation. One possibility is that there are some physiological changes, such as epigenetic changes, that arise due to mutagenesis, and which revert back to the wild type phenotype/physiological state in subsequent generations.

One example of such a reversion was the sickle shape seed phenotype in M_2 generation which had reverted back to wild type seed shape by the M5 generation.

Biotic stresses (insect pests and diseases) cause more than 42% of crop yield reduction in the world (Pimentel, 1997). Among the biotic stresses, Yellow stem borer, Sheath blight and BLB count amongst the important biotic stresses in rice. The resistance breeding for yellow stem borer has not gained any momentum due to the lack of suitable resistance/tolerance sources (Makkar and Bentur, 2017). Sheath Blight is another challenging disease due to unavailability of a reliable resistance source (Chen et al. 2019). Whereas, for management of BLB, there is an emergent need to widen the repertoire of resistance genes to enhance the spectrum and durability of resistance to *Xanthomonas oryzae pv. oryzae*. Mutagenesis in a gene may result in loss or gain in function with varying levels of expression and such types of mutations have prime importance while dealing with biotic stresses. Therefore, we attempted to identify resistance/tolerance stocks for YSB, Sheath blight and BLB from the mutagenized population. In a similar mutation study conducted by Wu et al. (2005), by irradiating IR64 with gamma rays, rice mutants that are resistant to Blast, BLB and tungro disease were identified. Similarly, Mohapatra et al. (2014) identified BB resistance mutants in an EMS induced Nagina 22 population. In the present study the identified biotic stress tolerance mutants under multi-location trials showed stability and durability of the mutants across different locations of India. Our study is consistent with the above-mentioned studies which indicate that mutagenized populations harbor a great amount of variability that can be recovered if the mutant population is subjected to appropriate screening techniques.

Earlier studies reported the frequency of EMS induced mutations within a range of 900–2000 and 2163–25753 in rice (Abe et al. 2012; Sevanthi et al. 2018). Our study revealed 2962-

12268 SNPs in the coding sequences (CDS). The exploitation of identified large number of SNPs by developing F_2 mapping populations can lead to identification of mutations responsible for the trait of interest. Evaluation of dis-similarity of the mutant lines with wild type using whole genome sequence analyses indicated that the mutants have diversity which ranged from 0.008% to 0.032% that indicate a range of variation.

Recent advances in high throughput sequencing technologies and computational approaches have facilitated the mapping of causal mutations in mutant lines (Austin et al. 2011; Schneeberger, 2014). In addition to gene identification, an allelic series comprised of INDELs can be useful in assessing gene function and for practical purposes of crop improvement. Therefore, comparison among the allelic series in SNP and INDELs across mutants and natural germplasm could help to infer the functional polymorphisms related to phenotypes observed in mutants and germplasm.

The mutants identified in this screen, especially those that have agronomically important phenotypes with significant similarity to the wild type could be useful to use in MutMap and QTL Seq for the identification of linked SNPs. Such studies will generate new knowledge and also have the potential for application in crop improvement.

Conclusions:

Exploiting genetic variability induced through mutations can be a powerful strategy in rice improvement where genetic gains may be diminishing due to a narrow gene pool. To generate variability, we have produced a large Samba Mahsuri mutant population that can be further employed in crop improvement. The huge demand for this variety and its wider adaptability and combining ability makes this mutant population a very useful resource for rice improvement. The value of these stocks will be enhanced with increasing usage and extensive

screening under a wide range of conditions. Systematic screens and mapping studies will not only help in generating new knowledge but will also help in translating the knowledge for crop improvement.

Declarations

Ethics approval and consent to participate

Not applicable

Consent for publication

Not applicable

Availability of data and material

Whole Genome Sequences reported in this article have been submitted to NCBI-SRA (BioProject ID: PRJNA658718).

Competing Interests

The authors declare that they have no competing interests.

Funding

Not applicable

Author's contribution:

RVS and MSM designed all experiments, developed the structure and arguments for the paper, made critical revisions, and approved the final version. PG, BV, BS and LS carried out the major work and prepared manuscript and contributed to the refinement of the manuscript. CGG, AK, KB, RM and HKP carried out whole genome sequencing and sequence analysis. JK and APP carried out the YSB screening. MGG, ME, GSL, MM, AS and BR helped in the screening of sheath blight and bacterial blight. LV, BCV and RV helped in the phenotypic evaluation of agro-

morphological traits. RMS helped in molecular work. All authors have read and approved the final manuscript.

Acknowledgements

The authors acknowledge the Council of Scientific and Industrial Research (CSIR), New Delhi, India and ICAR -IIRR for providing funds and infrastructure for carrying out the research work. RVS is supported by a J C Bose Fellowship from the Science and Engineering Research Board, Government of India.

Author's information

P. Gopi, Email: gopibiotech.1985@gmail.com

B. Vishalakshi, Email: vishalakshi.biotech@gmail.com

B. Suneel, Email: suneel.biotechnology@gmail.com

C.G. Gokulan, Email: gokulan@ccmb.res.in

A. Komal, Email: komal@ccmb.res.in

L. Swathi, Email: swathi.lvs@gmail.com

J. Karteek, Email: jallipalli.karteek@gmail.com

Md. Ershad, Email: ershad.cool@gmail.com

M. Milton, Email: milton2526@live.com

A. Srikanth, Email: srikanth.arutla977@gmail.com

B. Rajender, Email: rajender.burka007@gmail.com

GS. Laha, Email: lahags@yahoo.co.in

AP. Padmakumari, Email: padmakumariento@gmail.com

LV. Subbarao, Email: lvsubbarao1990@gmail.com

RM. Sundaram, Email: rms_28@rediffmail.com

B.C. Viraktamath, Email: mathrice@gmail.com

V. Ravindra Babu, Email: rbvemuri1955@gmail.com

B. Kranthi, Email: kranthi.brahma@ccmb.res.in

M. Raju, Email: raju.m@ccmb.res.in

HK. Patel Email: hitendrakpatel@gmail.com; hkpatel@ccmb.res.in

RV. Sonti, Email: sonti@ccmb.res.in

M. Sheshu Madhav, Email: sheshu24@gmail.com; sheshu_24@yahoo.com

Table captions:

Table 1. Number of mutants that exhibit agronomically important traits in each generation from M_2 to M_5 generation.

Table 2. Number of mutants that exhibit resistance against biotic stresses (YSB, BLB and Sheath Blight) from M_2 to M_5 generations.

Table 3. Sequence statistics of Samba Mahsuri (wild type), and mutant lines with different traits.

Table 4. Number of EMS-induced homozygous SNPs (GC to AT type), dissimilarity index, and the frequency of SNPs in the mutant lines with respect to SM.

Figure captions:

Figure 1. Distribution of mutants for various agro-morphological traits in M_2 generation.

Figure 2. A – M: Variants for different traits in the mutagenized population of Samba Mahsuri; W- Wild type Samba Mahsuri; M- Mutant; K1- Wild type with score 9; K2- Resistant mutant (SB 6) with score 1 and K3- Susceptible check TN 1 with score 9.

Supplementary data captions:

Supplementary Table 1: Primer sequence of all tested genes to detect allelic variations.

Supplementary Table 2: List of mutants that exhibit variations in morphological traits from M_2 to M_5 generations.

Supplementary Table 3: List of mutants that exhibit variations in physiological traits from M_2 to M_5 generations.

Supplementary Table 4: List of mutants that exhibit variations in panicle type from M_2 to M_5 generations.

Supplementary Table 5: List of mutants that exhibit variations in panicle emergence from M_2 to M_5 generations.

Supplementary Table 6: List of mutants that exhibit variations in yield attributing traits from M_2 to M_5 generations.

Supplementary Table 7: List of mutants that exhibit variations in grain types from M_2 to M_5 generations.

Supplementary Table 8: List of mutants that exhibit tolerance for YSB from M_2 to M_5 generations.

Supplementary Table 9: List of mutants that exhibit tolerance for sheath blight from M_2 to M_5 generations.

Supplementary Table 10: List of mutants that exhibit resistance for bacterial blight from M_2 to M_5 generations.

Supplementary Table 11: Screening of selected mutants with multiple isolates under multiple locations.

Supplementary Table 12: List of mutants that exhibit variations in multiple traits.

Supplementary Table 13: Allelic variations of mutants in comparison with reference allele and wild type allele among the tested genes.

Supplementary Table 14: Details of SNPs present in coding region of mutant lines.

Supplementary Figure 1: Related analysis using Tassel 5 indicated a high similarity among the mutant lines which is seen as single cluster containing majority of the mutant lines.

References:

Abe, A. *et al.* Genome sequencing reveals agronomically important loci in rice using MutMap. Nat Biotech, **30**, 174-178. https://doi.org/10.1038/nbt.2095 (2012).

Azad, M.A.K., Uddin, M.I. & Azam, M.A., Achievements in Rice research at BINA through Induced mutation. *Bioremediation, Biodiversity and Bioavailability.* **6**, 53-57 (2012).

Bhaktavatsalam, G., Satyanarayana, K., Reddy, A.P.K. & John, V.T. Evaluation for sheath blight resistance in rice. *Int Rice Res Newsletter.* **3**, 9-10(1978).

Bradbury, P.J., Zhang, Z., Kroon, D.E. and Casstevens, T.M., Ramdoss, Y. & Buckler, E.S. TASSEL: Software for association mapping of complex traits in diverse samples. *Bioinformatics*. **23**, 26332635. doi: 10.1093/bioinformatics/btm308 (2007).

Brooks, S.A., Yan, W., Jackson, A.K. & Deren, C.W. A natural mutation in rc reverts white-rice-pericarp to red and results in a new, dominant, wild-type allele: Rc-g. *Theoretical and applied genetics.* **117**, 575-580. doi: 10.1007/s00122-008-0801-8 (2008).

Chakrabarti, S.N. Mutation breeding in India with particular reference to PNR rice varieties. *Journal of Nuclear Agriculture and Biology.* **24**, 73-82 (1995).

Chen, Z. *et al.* Identification of new resistance loci against sheath blight disease in rice through genome-wide association study. *Rice Science.* **26**(1), 21-31. doi: 10.1016/j.rsci.2018.12.002 (2019).

Cingolani, P. *et al.* A program for annotating and predicting the effects of single nucleotide polymorphisms, SnpEff: SNPs in the genome of Drosophila melanogaster strain w1118; iso-2; iso-3. *Fly*. **6**, 80-92. doi: 10.4161/fly.19695 (2012).

Danecek, P. *et al.* The variant call format and VCFtools. *Bioinformatics.* **27**, 2156-2158. doi: 10.1093/bioinformatics/btr330 (2011).

Devasena, N., Soundararajan, R.P., Reuolin, S.J., Jeyaprakash. P. & Robin, S. Evaluation of Rice Genotypes for Resistance to Yellow Stem borer, Scirpophaga incertulas (Walker) through Artificial Screening Methods. *JEZS.* **6**, 874-87(2018).

Du, H. *et al.* Sequencing and de novo assembly of a near complete indica rice genome. *Nature communications.* **8**, 1-12. doi: 10.1038/ncomms15324(2017).

Fu,Y. Understanding crop genetic diversity under modern plant breeding. *Theor Appl Genet*. **128**, 2131–2142. doi: 10.1007/s00122-015-2585-y (2015).

Garrison, E. & Marth, G. Haplotype-based variant detection from short-read sequencing. *arXiv*, arXiv:**1207**, 1-9 (2012).

Hai, L. Quantitative trait loci (QTL) of stem strength and related traits in a doubled-haploid population of wheat (Triticum aestivum L.). *Euphytica*. **141,** 1-9. doi: 10.1007/s10681-005-4713-2(2005).

Hajira SK, Sundaram RM et al A Single-tube, functional marker-based multiplex PCR assay for simultaneous detection of major bacterial blight resistance genes in rice, Xa21, xa13 and xa5. Rice Sci 23:1224–1227(2016).

IRRI. Standard Evaluation System for Rice (4th edn), International Rice Research Institute, Manila, the Philippines (1996).

IRRI. Standard Evaluation System for Rice. International Rice Research Institute, Manila, the Philippines (2014).

Jiang, L. *et al.* Whole-genome sequencing identifies a rice grain shape mutant, gs9–1. *Rice.* **12**, 52 doi: 10.1186/s12284-019-0308-8 (2019).

Kauffman, H. E., Reddy, A.P.K., Hsieh, S.P.Y. & Merca, S.D. An improved technique for evaluating resistance of rice varieties to Xanthomonas oryzae. Plant Dis. Rep. **57**, 537-541(1973).

Kharkwal, M.C. & Shu, Q.Y. The role of induced mutations in world food security. Shu QY (eds) In: Induced plant mutations in the genomics era. *Food and Agriculture Organization of the United Nations, Rom.* 33–38 (2009)

Krishnan, A. *et al.* Mutant resources in rice for functional genomics of the grasses. Plant Physiol. **149**, 165–170. doi: 10.1104/pp.108.128918 (2009).

Lee, J., Lee, A.R. & Kwon, S.W. Effect of proton beam irradiation on M1 seeds and seedling growth in rice. *Plant Breeding and Biotechnology* **3**, 384–388. doi:10.9787/PBB.2015.3.4.384 (2015).

Li, G. *et al.* The sequences of 1504 mutants in the model rice variety Kitaake facilitate rapid functional genomic studies. *The Plant Cell*. **29,** 1218-1231. doi: 10.1105/tpc.17.00154 (2017).

Li, T., Liu, B., Spalding, M.H., Weeks, D.P. & Yang, B. High-efficiency TALEN-based gene editing produces disease-resistant rice. *Nature biotechnology*. **30**, 390. doi: 10.1038/nbt.2199 (2012).

Makkar, G.S. & Bentur, J.S. Breeding for Stem Borer and Gall Midge Resistance in Rice. In: R. Arora, S. Sandhu (eds) Breeding Insect Resistant Crops for Sustainable Agriculture. *Springer, Singapore.* 323-352. *doi: 10.1007/978-981-10-6056-4_11* (2017).

McCallum, C.M., Comai, L., Greene, E.A. & Henikoff, S. Targeting Induced Local Lesions in Genomes (TILLING) for plant functional genomics. *Plant Physiol*. **123**, 439–442. doi: 10.1104/pp.123.2.439 (2000).

Mohapatra, T. et al. EMS induced mutants of upland rice variety Nagina22: generation and characterization. *Proc Indian Natl Sci Acad*. **80**, 163-172. doi: 10.16943/ptinsa/2014/v80i1/55094 (2014).

Morita, R., Kusaba, M., Iida, S., Yamaguchi, H., Nishio, T. & Nishimura, M. Molecular characterization of mutations induced by gamma irradiation in rice. *Genes & genetic systems,* **84**, 361-370. doi: 10.1266/ggs.84.361 (2009).

Mustikarini, E.D., Ardiarini, N.R., Basuki, N. & Kuswanto, K. The improvement of early maturity red rice mutant trait for drought tolerance. *International Journal of Plant Biology,* **7**. doi: 10.4081/pb.2016.6345 (2016).

Okuno, K. & Kawai, T. Variations of internode length and other characters in induced long-culm mutants of rice. *Jpn J Breed.* **28**, 243-250. doi: 10.1270/jsbbs1951.28.243 (1978).

Oladosu, Y. *et al.* Principle and application of plant mutagenesis in crop improvement: a review. *Biotechnology & Biotechnological Equipment*. **30**,1-16, doi: 10.1080/13102818.2015.1087333 (2016).

Ookawa, T. *et al.* New approach for rice improvement using a pleiotropic QTL gene for lodging resistance and yield. *Nature communications*. **1**,1-11. https://doi.org/10.1038/ncomms1132 (2010).

Ookawa, T. *et al.* Precise estimation of genomic regions controlling lodging resistance using a set of reciprocal chromosome segment substitution lines in rice. *Sci Rep.* **6,** 1-12. doi: 10.1038/srep30572 (2016).

Pathak, H., Nayak, A.K., Jena, M., Singh, O.N., Samal, P. & Sharma, S.G. Rice Research for Enhancing Productivity, Profitability and Climate Resilience (2018).

Pimentel, D. Pest management in agriculture. In Pimentel D (Ed) Techniques for Reducing Pesticide Use: Economic and Environmental Benefits. Chichester, United Kingdom: *Wiley*. 1–11(1997).

Ram, H., Soni, P., Salvi, P., Gandass, N., Sharma, A., Kaur, A. & Sharma, T.R. Insertional mutagenesis approaches and their use in rice for functional genomics. *Plants*. **8**, 310. doi: 10.3390/plants8090310 (2019).

Ramkumar, G. *et al.* (2010) Development of a PCR-based SNP marker system for effective selection of kernel length and kernel elongation in rice. *Mol Breeding.* **26**,735–740. doi: 10.1007/s11032-010-9492-3 (2010).

Ramkumar, M.K. A novel stay-green mutant of rice with delayed leaf senescence and better harvest index confers drought tolerance. *Plants.* **8**,375. doi: 10.3390/plants8100375 (2019).

Rao, D.R.M. & Reddi, T.S. Azide mutagenesis in rice. Proceedings: *Plant Sciences*. **96**, 205-215 (1986).

Rencui, Y., Shubiao, Z. & Ronghua, H. Breeding technology of eui-hybrids of rice. *Zhongguo Nongye Kexue (China)*. **1,** 359-363 (2002).

Roy, B., Kumar, V., Tulsiram, S.D. & Das, B.K. Development of high yielding aromatic mutants of rice (Oryza sativa L.) from a local aromatic cultivar, Tulaipanji by using gama radiation. *Indian J. Genet*. **78**, 409-416. doi: 10.31742/IJGPB.78.4.2 (2018).

Ruengphayak, S., Ruanjaichon, V., Saensuk, C., Phromphan, S., Tragoonrung, S., Kongkachuichai, R. & Vanavichit, A. 2015. Forward screening for seedling tolerance to Fe toxicity reveals a polymorphic mutation in ferric chelate reductase in rice. *Rice*. **8**, 3. doi: 10.1186/s12284-014-0036-z (2015).

Rutger, J.N. & Carnahan, H.L. 1981. A fourth genetic element to facilitate hybrid cereal production—A recessive tall in rice 1. *Crop Science*. **21**, 373-376. doi: 10.2135/cropsci1981.0011183X002100030005x (1981).

Sakamoto, T. *et al*. Brassinosteroidinduced bending of the leaf lamina of dwarf rice seedlings: an auxin-mediated phenomenon. *Nat Biotechnol*. **24**, 105-109. doi: 10.1038/nbt1173 (2006).

Schneeberger, K. Using next-generation sequencing to isolate mutant genes from forward genetic screens. *Nat Rev Genet.* **15**, 662-676. doi: 10.1038/nrg3745 (2014).

Sevanthi, A. *et al.* 2018. Whole genome characterization of a few EMS-induced mutants of upland rice variety Nagina 22 reveals a staggeringly high frequency of SNPs which show high phenotypic plasticity towards the wild-type. *Frontiers in plant science*. **9**, 1179. doi: 10.3389/fpls.2018.01179 (2018).

Shen, Z.T. Studies on eliminating panicle enclosure in WA type MS line of rice Oryza sativa subsp. indica. *Chin J Rice Sci.* **1**,95-99 (1987).

Shoba, D, Raveendran M *et al* Development and genetic characterization of a novel herbicide Imazethapyr) tolerant mutant in rice (Oryza sativa L.) Rice 10(1):10. doi: 10.1186/s12284-017-0151-8. (2017)

Singh, N. *et al.* 2016. Genetic diversity trend in Indian rice varieties: an analysis using SSR markers. *BMC genetics.* **17**, 127. doi: 10.1186/s12863-016-0437-7 (2016)

Singh, V.K., Ellur, R.K., Singh, A.K., Nagarajan, M., Singh, B.D. & Singh, N.K. Effect of qGN4. 1 QTL for grain number per panicle in genetic backgrounds of twelve different mega varieties of rice. *Rice*, **11**, 8. doi: 10.1186/s12284-017-0195-9 (2018).

Soomro, A.M., Baloch, A.W., Ahmed, M., Javed, M.A., Bughio, H.R., Bughio, M.S., Muhammad, T. & Mastoi, N.N. Evaluation of Induced Mutants of Rice for Yield and Quality Characters. *Asian Journal of Plant Sciences.***1**, 248-249. doi: 10.3923/ajps.2002.248.249 (2002).

Sundaram, R.M. 2008. Marker assisted introgression of bacterial blight resistance in Samba Mahsuri, an elite indica rice variety. *Euphytica.* **160**, 411-422 (2008).

Sundaram, R.M. Multi-trait improvement in rice through marker-assisted breeding. *ORYZA-An International Journal on Rice.* **55,** 24-37 (2018).

Suzuki, T. MNU-induced mutant pools and high performance TILLING enable finding of any gene mutation in rice. *Molecular Genetics and Genomics*. **279**, 213-223. doi: 10.1007/s00438-007-0293-2 (2008).

Taguchi-Shiobara, F. Natural Variation in the Flag Leaf Morphology of Rice Due to a Mutation of the NARROW LEAF 1 Gene in Oryza sativa L. *Genetics*. **201,** 795-808. doi: 10.1534/genetics.115.181040 (2015).

Tai, T.H., Chun, A., Henry, I.M., Ngo, K.J. & Burkart-Waco, D. Effectiveness of sodium azide alone compared to sodium azide in combination with methyl nitrosurea for rice mutagenesis. *Plant Breeding and Biotechnology*. **4,** 453-461. doi: 10.9787/PBB.2016.4.4.453 (2016).

Takai, T. 2013. A natural variant of NAL1, selected in high-yield rice breeding programs, pleiotropically increases photosynthesis rate. *Scientific reports*. **3**, 2149. doi: 10.1038/srep02149 (2013)

Talebi, A.B., Talebi, A.B. & Shahrokhifar, B. Ethyl methane sulphonate (EMS) induced mutagenesis in Malaysian rice (cv. MR219) for lethal dose determination. *American Journal of Plant Sciences*: **3**, 1661. doi: 10.4236/ajps.2012.312202 (2012).

Till, B.J. *et al*. Discovery of chemically induced mutations in rice by TILLING. *BMC plant biology*. ***7***, 19. doi: 10.1186/1471-2229-7-19 (2007).

Viana, V.E., Pegoraro, C., Busanello, C. & de Oliveira, A.C. 2019. Mutagenesis in rice: the basis for breeding a new super plant. *Frontiers in plant science*. **10,** 1-28. doi: 10.3389/fpls.2019.01326 (2019).

Wang, N., Long, T., Yao, W., Xiong, L., Zhang, Q. & Wu, C. Mutant resources for the functional analysis of the rice genome. *Molecular plant.* **6**, 596-604. doi: 10.1093/mp/sss142 (2013).

Wu, J.L. *et al.* Chemical-and irradiation-induced mutants of indica rice IR64 for forward and reverse genetics. *Plant molecular biology*, **59**, 85-97. doi: 10.1007/s11103-004-5112-0 (2005).

Xie, K., Minkenberg, B. & Yang, Y. Boosting CRISPR/Cas9 multiplex editing capability with the endogenous tRNA-processing system. *Proceedings of the National Academy of Sciences.* **112**, 3570-3575. https://doi.org/10.1073/pnas.1420294112 (2015).

Xu, R. *et al*. Generation of targeted mutant rice using a CRISPR-Cpf1 system. *Plant biotechnology journal*. **15,**713-717. doi: 10.1111/pbi.12669 (2017).

Xu, Y.H. *et al*. Identification of a 98-kb DNA segment containing the rice Eui gene controlling uppermost internode elongation, and construction of a TAC transgene sublibrary. *Molecular Genetics and Genomics*. **272,** 149-155. doi: 10.1007/s00438-004-1019-3 (2004).

Xuan, Y.H. *et al.* Transposon Ds-Mediated Insertional Mutagenesis in Rice (Oryza sativa). *Current protocols in plant biology*. **1,** 466-487. doi: 10.1002/cppb.20030 (2016)

Yadav, S. *et al.* 2017. Molecular mapping of QTLs associated with lodging resistance in dry direct-seeded rice (Oryza sativa L.). *Frontiers in plant science.* **8**, 1431. doi: 10.3389/fpls.2017.01431 (2017)

Yuan, L.P. & Virmani, S.S. Status of hybrid rice research and development. *Hybrid Rice IRRI.* 7-24. (1998)

Yugander, A. *et al.* Incorporation of the novel bacterial blight resistance gene Xa38 into the genetic background of elite rice variety Improved Samba Mahsuri. *Plos one*.**13**, 1-16. doi: 10.1371/journal.pone.0198260 (2018).

Table 1: Details (number) of mutants that exhibit agronomically important traits from M_2 to M_5 generation.

S. No.	Character	M_2	M_3	M_4	M_5^*		
						Range	
						Mutant	Wild type
**I.**	**Morphological Characters**						
i.	Tall plant	43	12	10	7	154-139cm	97.5cm
ii.	Dwarf	36	4	2	1	90cm	97.5cm
iii.	Bonsai (grassy)	1	1	1	1	35cm	97.5cm
iv.	Pink apicules	3	3	1	1	Present	Absent
	**Total (I)**	**83**	**20**	**14**	**10**		
**II.**	**Physiological Characters**						
i.	Flag leaf variations (Broad & Long)	28	18	15	9	L- 45.5-48.5cm; W-2.2- 3.1cm	L- 28.2cm W- 1.3cm
ii.	Leaf variations (Narrow)	7	4	4	1	L- 25.3cm W-0.8cm	Absent
iii.	Albino	5	-	-	-	Present	Absent
iv.	Xantha	5	-	-	-	Present	Absent
v.	Coloration	11	-	-	-	Present	Absent
vi.	Strong culm (Culm strength)	26	17	17	17	26-32N/m^2	25N/m^2
vii.	Maturity (Early flowering)	73	36	36	36	108-132days	135days
viii.	Stay green	4	3	2	2	Present	Absent
ix.	Sterile plant	28	-	-	-	Present	Absent
x.	Shattering	4	3	2	2	Present	Absent
	**Total (II)**	**191**	**81**	**76**	**67**		
**III.**	**Panicle Type**						
i.	Sparse Panicle	5	3	2	2	Present	Absent
ii.	Compact/Dense Panicle	30	13	8	5	Present	Absent
iii.	Long Panicle	50	32	28	15	30-32cm	15.6cm
	**Total (III)**	**85**	**48**	**38**	**22**		
**IV.**	**Panicle Emergence**						
i.	Elongation of Upper Internode (EUI)	27	11	11	9	7-26cm	Absent
ii.	Complete panicle emergence	155	31	22	13	2.0-2.5cm	Absent
	**Total (IV)**	**182**	**42**	**33**	**22**		
**V.**	**Yield**						
i.	More number of tillers	13	9	8	5	15-26	12

ii.	More productive tillers & better grain filling	484	120	22	8	13-25	12
iii.	High grain number	15	3	2	2	221-306 g/p	200g/p
iv.	Phenotypic acceptability	20	8	4	2	Present	Absent
	**Total (V)**	**532**	**140**	**36**	**17**		
**VI.**	**Grain types**						
i.	Long slender grain without awns	32	18	18	7	L-12.6-13.0mm<br>B- 2.2-2.5mm	Absent
ii.	Long slender grain with awns	11	11	10	3	L-24-29mm<br>B- 2.2-2.5mm	Absent
iii.	Long bold grain with awns	2	2	1	1	L-19mm<br>B-3.2mm	Absent
iv.	Long bold grain without awns	27	16	14	10	L-10.2-12.2mm<br>B-3.1-3.5mm	Absent
v.	Medium slender grain with awns	4	4	3	1	L-9.8mm<br>B-2.3mm	Absent
vi.	Medium bold grain with awns	2	2	1	1	L-9.9mm<br>B-3.4mm	Absent
vii.	Medium bold grain without awns	38	22	20	15	L-8.9-9.0mm<br>B-3.3-3.5mm	Absent
viii.	Short bold	13	4	4	2	L-4.8-5.0mm<br>B-3.2-3.5mm	Absent
ix.	Short slender	22	5	5	2	L-5.1-5.4mm<br>B-2.3-2.4mm	Absent
x.	Sickle shape	7	3	3	-	Present	Absent
	**Total (VI)**	**158**	**87**	**79**	**42**		
	**Total (I+II+III+IV+V+VI)**	**1231**	**418**	**276**	**180**		

*L-Length; B- Breadth; W- Width; g/p-grains per panicle; * indicates the only selected and non segregating mutants*

Table 2 Details of mutants (numbers) that exhibit resistance against biotic stresses (YSB, BLB and Sheath Blight) from M_2 to M_5 generations.

S. No	Trait	M_2	M_3	M_4	M_5
1.	Yellow Stem Borer (YSB)	170	30	10	08
2.	Bacterial Leaf Blight (BLB)	482	261	40	13
3.	Sheath Blight (SB)	801	620	40	13
	**Total**	**1453**	**1181**	**90**	**34**

Table 3: Sequence statistics of Samba Mahsuri (wild type) and mutant lines with different traits

S. No.	Line	Type or trait of Line	Number of Raw Reads (R1+R2)	QC Passed Reads (R1+R2)	Sequenced bases (Gb)	Coverage (X)	Mapped QC passed Reads (R1+R2)	Mapped Reads (%)	Reference used
**1**	**SM-E**	Wildtype	112285084	102241208	15.34	39.22	100885182	98.67	**R498**
**2**	**SM-G**	Wildtype	110937222	111916395	16.79	42.94	110657624	98.88	
**3**	**SM-C**	Wildtype	123573016	111003694	16.65	42.59	122513940	98.27	
**4**	**TI-109**	Complete Panicle Exsertion	114514030	114191395	17.13	43.81	103577844	98.73	**Samba Mahsuri**
**5**	**TI-110**	Complete Panicle Exsertion	107814208	96892528	14.53	37.17	96892528	98.77	
**6**	**TI-128**	High Yield; High Spikelets per Panicle	113249162	102101092	15.32	39.17	102101092	98.89	
**7**	**TI-35**	Elongated Uppermost Internode	108635160	108364345	16.25	41.57	97603326	98.76	
**8**	**TI-38**	Complete Panicle Exsertion	113152500	102614244	15.39	39.37	102614244	98.86	
**9**	**TI-42**	Bacterial Blight Tolerance	106403198	78348650	11.75	30.06	78348650	98.62	
**10**	**TI-50**	High Yield; High Spikelets per Panicle	109924526	99290257	14.89	38.09	99290257	98.90	
**11**	**SB-6**	Sheath Blight Tolerance	126418290	125999511	18.90	48.34	112679044	98.62	

Table 4: Details of SNPs in the mutant lines with respect to wild type

S. No.	Line	Trait	Number of SNPs	Dissimilarity index (%)[a]	SNPs per Mb
**1**	**TI-109**	Complete Panicle Exsertion	69765	0.018	178.4
**2**	**TI-110**	Complete Panicle Exsertion	59320	0.015	151.7
**3**	**TI-128**	High Yield; High Spikelets per Panicle	126019	0.032	322.3
**4**	**TI-35**	Elongated Uppermost Internode	34121	0.009	87.3
**5**	**TI-38**	Complete Panicle Exsertion	65556	0.017	167.7
**6**	**TI-42**	Bacterial Blight Tolerance	29850	0.008	76.3
**7**	**TI-50**	High Yield; High Spikelets per Panicle	124933	0.032	319.5
**8**	**SB-6**	Sheath Blight Tolerance	37387	0.010	95.6

figure-1 Click here to access/download;Figure;Figure 1.jpg

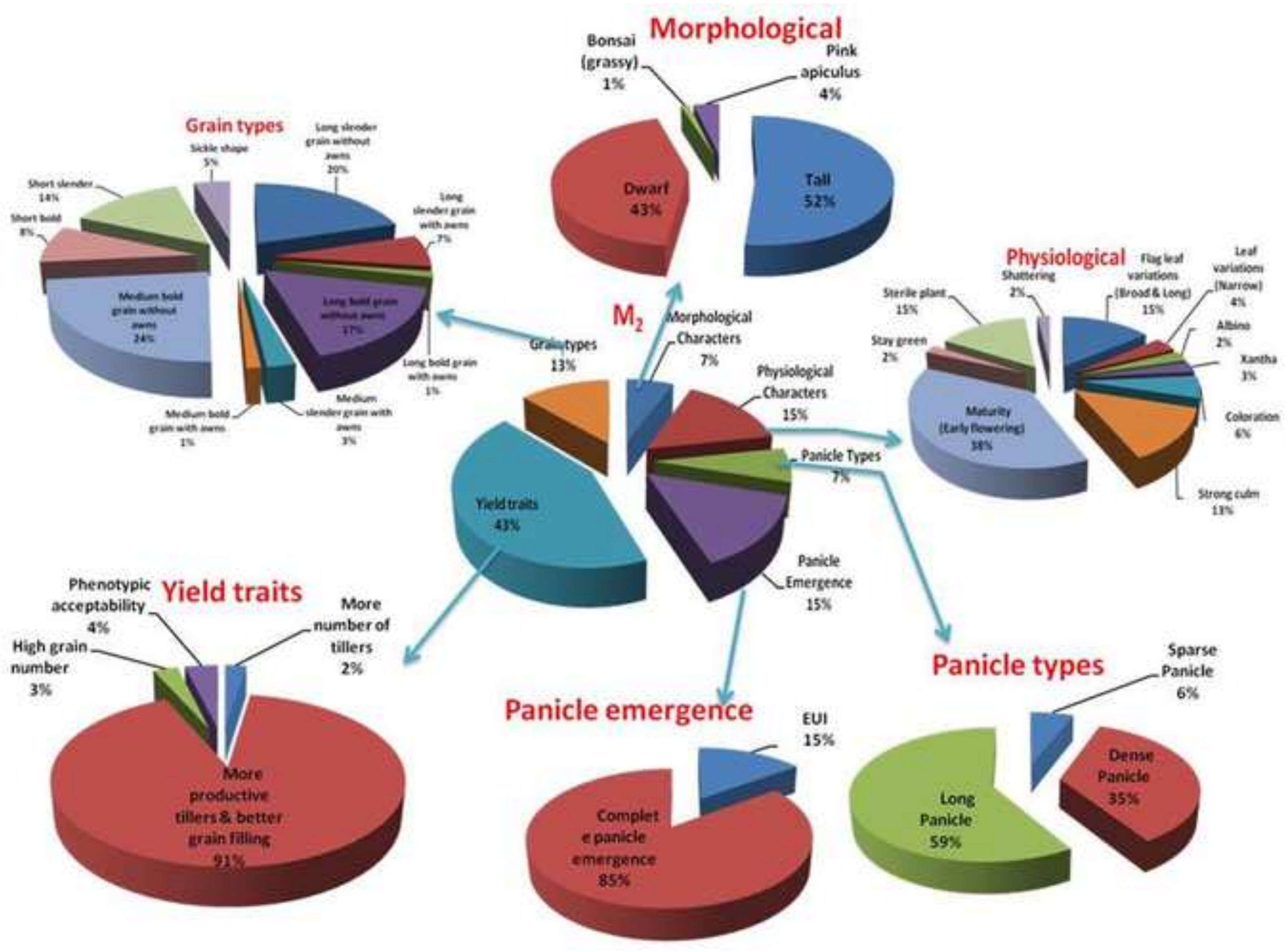

Figure 1. Distribution of mutants for various agro-morphological traits in M_2 generation

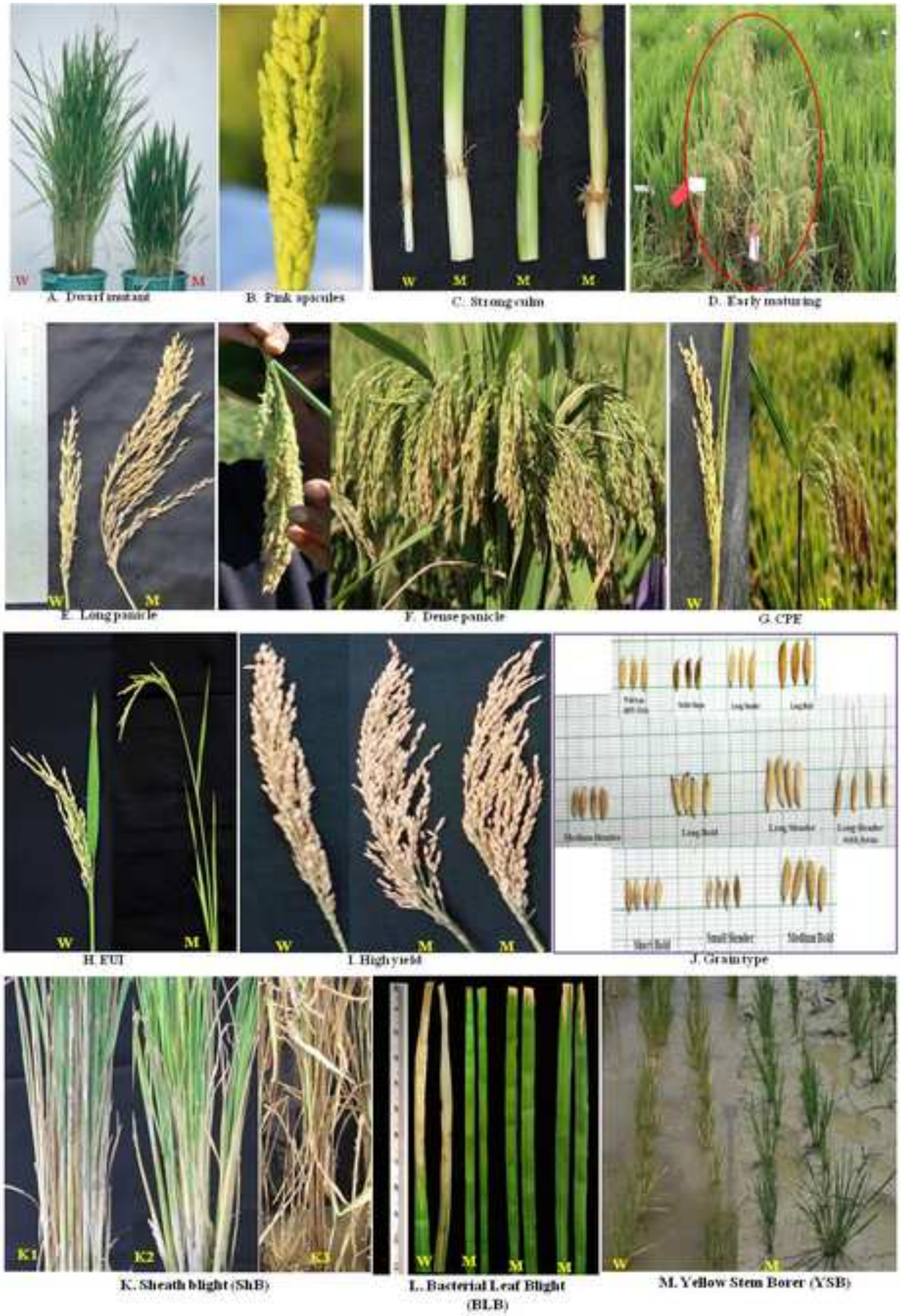

Figure 2. A – M: Variants for different traits in the mutagenized population of Samba Mashuri ; W- wild type Samba Mashuri ; M- Mutant; K1- Wild type with score 9; K2- Resistant mutant (SB 6) with score 1 and K3- Susceptible check TN 1 with score 9.

supplementary figure-1

Click here to access/download
Supporting Information
Figure S1.jpg

supplementary table-1

Click here to access/download
Supporting Information
Table S1_Allele mining Primers.xls

supplementary table-2

Click here to access/download

Supporting Information

Table S2_M2 to M5-Morphological data.xlsx

supplementary table-3

Click here to access/download
Supporting Information
Table S3_M2 to M5-Physiological data.mod.xlsx

supplementary table-4

Click here to access/download

Supporting Information

Table S4_M2 to M5-Panicle type data.xlsx

supplementary table-5

Click here to access/download
Supporting Information
Table S5_M2 to M5-Panicle emergence data.xlsx

supplementary table-6

Click here to access/download
Supporting Information
Table S6_M2 to M5-Yield data.xlsx

Click here to access/download
Supporting Information
Table S7_M2 to M5-Grain type data.xlsx

supplementary table-8

Click here to access/download
Supporting Information
Table S8_M2 to M5-YSB data.xls

supplementary table-9

Click here to access/download
Supporting Information
Table S9_M2 to M5-Sheath blight data.xls

Click here to access/download
Supporting Information
Table S10_M2 to M5-Bacterial blight data.xls

Click here to access/download
Supporting Information
Table S11_Multiple isolates data.xls

supplementary table-12

Click here to access/download
Supporting Information
Table S12_Multiple trait data.xls

supplementary table-13

Click here to access/download

Supporting Information

Table S13_SNPs data in mutants.xls
