# Supplementary material for: Mutation resource of Samba Mahsuri revealed the presence of high extent of variations among key traits for rice improvement
Source: PLoS One. 2021 Oct 20;16(10):e0258816. doi: 10.1371/journal.pone.0258816 (PMC8528289; doi:10.1371/journal.pone.0258816)
Supplement: Attachment — Submitted filename: Referees comment.doc [file pone.0258816.s015.doc]

To

Chief Editor

PLOS ONE

Dear Sir,

I have made some of the following remarks about the manuscript entitled “Mutation resource of Samba Mahsuri revealed the presence of high extent of variations among key traits for rice improvement”.

1. This article is contributable to science & technology if improvements can be made.
2. As the author is concerned about efficient development of genetic stock through mutagenesis.
3. Title as well as the objectives of the research article are nicely reflected the manuscript.
4. Authors need to follow the exact format of the POLS ONE journal in the text as well as in the reference sections of the manuscript.
5. References should be thoroughly checked and written in the text as well as in the References section as per Journal.
6. Paper required a revision to follow Journal format.

With regards,

R. K. Salgotra

Professor & Coordinator

School of Biotechnology

SKUAST-J, Chatha, Jammu (J&K)

INDIA
