# Supplementary material for: Mutation resource of Samba Mahsuri revealed the presence of high extent of variations among key traits for rice improvement
Source: PLoS One. 2021 Oct 20;16(10):e0258816. doi: 10.1371/journal.pone.0258816 (PMC8528289; doi:10.1371/journal.pone.0258816)
Supplement: Attachment — Submitted filename: Response to Reviewer comments.docx [file pone.0258816.s017.docx]

**Response to Reviewers**

Thank you for giving us the opportunity to submit a revised draft of the manuscript “Mutation resource of Samba Mahsuri revealed the presence of high extent of variations among key traits for rice improvement” for publication in the Plos One journal. We appreciate the time and effort that you and the reviewers dedicated to providing feedback on our manuscript and are grateful for the insightful comments on and valuable improvements to our paper. We have incorporated the suggestions made by the reviewers.

**Reviewers’ comments to the authors:**

**Reviewer #1:**

1. This article is contributable to science & technology if improvements can be made.

**Reply:** Thank you. Authors improved the manuscript to meet the journal standards.

2. As the author is concerned about efficient development of genetic stock through mutagenesis.

**Reply:** Yes we are very much concerned to develop lines possessing biotic stresses (YSB, ShB and BLB) tolerance as well as several economically important traits through mutagenesis. These unique genetic resources can be utilized for the identification of novel genes/alleles for different traits. The lines which have better agronomic features can be used as pre-breeding lines.

3. Title as well as the objectives of the research article are nicely reflected the manuscript.

**Reply:** Thank you. We appreciate the time and effort that you and the reviewers have dedicated to providing your valuable feedback on our manuscript

4. Authors need to follow the exact format of the POLS ONE journal in the text as well as in the reference sections of the manuscript.

**Reply:** Thank you for this suggestion; we have edited the manuscript and reference in PLOS ONE journal format.

5. References should be thoroughly checked and written in the text as well as in the References section as per Journal.

**Reply:** Thank you for pointing this out. We have checked all the reference and revised to journal format

6. Paper required a revision to follow Journal format.

**Reply:** We agree with this comment. We revised the manuscript in PLOS ONE journal format

**Reviewer #2:**

i. In Table S6, please provide comparative data of mutants at M4 or M5 generation with the wild type for the trait, high grain number using statistical parameters.

**Reply:** Thank you for valuable suggestion. We performed the statistical analysis (ANOVA) of grain number of mutant and wild type and the data is mentioned in Table S6

ii. In Table S8, provide the YSB score of mutants at M4 generation with wild type.

**Reply**: Thank you for this suggestion. YSB phenotypic score of wild type (Samba Mahsuri) was mentioned at M4 generation

iii. In Table S12, provide comparative data of mutants at M4 or M5 generation with the wild type for the trait, yield/plant using statistical parameters.

**Reply:** As suggested by the reviewer the statistical analysis (ANOVA) was performed for yield/plant and the data is mentioned in Table S12.

iv. Check English language

**Reply**: Thank you for the valuable suggestion. For improvement of English, we have given manuscript to our institute science editor and improved substantially
